# Supplementary material for: COBRA improves the completeness and contiguity of viral genomes assembled from metagenomes
Source: Nat Microbiol. 2024 Feb 6;9(3):737–50. doi: 10.1038/s41564-023-01598-2 (PMC10914622; doi:10.1038/s41564-023-01598-2)
Supplement: Supplementary file 1 — Supplementary Figs. 1–12 and Supplementary Results and Discussion. [file 41564_2023_1598_MOESM1_ESM.pdf]

nature microbiology

Article https://doi.org/10.1038/s41564-023-01598-2

COBRA improves the completeness and contiguity of viral genomes assembled from metagenomes

In the format provided by the authors and unedited

**Contents**	**Title**
Supplementary results and discussion	The simulation of genomes with repeats and variations.
	COBRA accurately joins viral sequences from a simulated soil dataset.
	Performance comparison of COBRA against ContigExtender.
	Expansion of the diversity of actinophages with *whiB* family transcriptional regulators.
Supplementary Figure 1	Simulated sequences with intra-genome repeats shorter than the largest kmer used in *de novo* assembly.
Supplementary Figure 2	Simulated sequences with intra-genome repeats that are longer than the largest kmer used in *de novo* assembly.
Supplementary Figure 3	Simulated sequences with inter-genome shared regions.
Supplementary Figure 4	Simulated sequences with variations between two subpopulations.
Supplementary Figure 5	Simulated sequences with variations among three subpopulations.
Supplementary Figure 6	Circular extension of query virus contigs by COBRA.
Supplementary Figure 7	One "extended_circular" from the MEGAHIT assembly with < 90% "AF_COBRA" and < 90% "AF_polished".
Supplementary Figure 8	Examples of COBRA and polished genome pairs with relatively lower "AF_COBRA" values.
Supplementary Figure 9	Freshwater ecosystem genomes broaden the diversity of *whiB*-encoding actinophages.
Supplementary Figure 10	Examples of the *in situ* gene expression of whiB-encoding actinophages with genomes reconstructed from Lake Rotsee.
Supplementary Figure 11	Evidence indicating that the two published huge phages with genome sizes over 800 kbp were prophage sequences.
Supplementary Figure 12	The number of sulfur metabolism-related AMGs detected in the phage genomes.

Supplementary results and discussion

The simulation of genomes with repeats and variations.

To test how metaSPAdes, IDBA_UD, and MEGAHIT assemble short reads from genomes with **intra-genome repeat regions** (including direct terminal repeats, DTRs), **inter-genome shared regions**, and **within-population variation** (Supplementary Table 1), we obtained simulated genomes for each case. We generated in-silicon Illumina HiSeq paired-end reads (126 bp X 2) using InSilicoSeq (Gourlé et al. 2019). The reads were *de novo* assembled using the three assemblers individually. The assembled results were compared against the simulated genomes, and the number of contigs from the assemblies, and the end sequences between contigs were analyzed for overlaps. See below for details of each simulated case.

Intra-genome repeats. We obtained simulated genomes with a region that repeats twice and three times, at both distant (means the repeat regions are distant from each other) and consecutive circumstances (means the repeat regions are next to each other) (Supplementary Fig. 1, Supplementary Table 1).

If the repeat length is shorter than the maxK used in assembly (Supplementary Fig. 1, Supplementary Table 1), only two assemblies generated fragmented contigs, (1) three times repeats that are distant from each other in the simulated genome, and assembled by MEGAHIT, and (2) three times of repeats that are consecutive to each other in the simulated genome, and assembled by IDBA_UD. For all the other cases, only one contig was generated in each, however, MEGAHIT sometimes generated only one copy of the consecutive repeats.

If the repeat length is longer than the maxK used in assembly (Supplementary Fig. 2, Supplementary Table 1), the assemblers usually generated fragmented contigs excepting, (1) two times repeats that are distant from each other in the simulated genome, and assembled by metaSPAdes, (2) two times of repeats that are consecutive to each other, and assembled by all three assemblers, while only metaSPAdes got the correct copy number of the repeats, and (3) three times of repeats that are consecutive to each other, and assembled by MEGAHIT, while with only one copy of the repeat in the contig. In all the other assemblies, two or three contigs were generated, and the length of the contig end sequences between contigs is expected (i.e., maxK or maxK-1).

We also obtained simulated genomes with DTR shorter than maxK or longer than maxK (Supplementary Table 1). We found no matter whether the DTR length is longer or shorter than the maxK, (1) if the genome did not contain another intra-genomic repeat longer than the maxK, all three assemblers will generate a single contig. (2) If the genome contains another intra-genomic repeat longer than the maxK, metaSPAdes will still generate a single contig, while IDBA_UD and MEGAHIT will obtain three contigs that broke at the intra-genomic repeat sites.

Inter-genome shared regions. We obtained two simulated genomes (genome 1 and 2) with a shared region longer than maxK in two cases, (1) coverage of genome 1 = 10 X, coverage of genome 2 = 30 X (Supplementary Fig. 3, upper panel), and (2) both genomes have a coverage of 20 X (Supplementary Fig. 3,

bottom panel). In case (1), both IDBA_UD and MEGAHIT obtained five contigs, with the shared region represented by a single contig of 40 X coverage, and it could be used to concatenate the two contigs from genome 1 into a whole genome, and also the two contigs from genome 2 into a whole genome, and the contig end sequences are with an overlap of the expected length (maxK-1 for IDBA_UD and maxK for MEGAHIT), while metaSPAdes obtained three contigs, one of them represented the whole sequence of genome 2 (the dominant one), and the other two contigs represented the two parts of the genome 1 without the shared region. In case (2), IDBA_UD and MEGAHIT obtained the same contigs as in case (1), while metaSPAdes obtained the complete sequences of both genomes. These simulated results indicated that metaSPAdes is doing better than the other two assemblers in such cases.

We did not show here the simulated genomes with a shared region shorter than the maxK, as in this case, all three assemblers will obtain two complete genomes without fragmentation.

Within-population variations. For within-population variations, we obtained simulated genomes of two subpopulations (Supplementary Fig. 4) and three subpopulations (Supplementary Fig. 5).

For two subpopulations, if the variation region is shorter than maxK, a contig representing the more abundant subpopulation was generated by all three assemblers (Supplementary Fig. 4a). If the variation region is longer than maxK, IDBA_UD always breaks at the starting and ending points of the variable regions (Supplementary Fig. 4b). However, metaSPAdes and MEGAHIT generated different numbers of contigs that are dependent on the abundance of the subpopulations (reflected by the coverage of the simulated genomes), they generated fragmented contigs as IDBA_UD did when the abundance of the two subpopulations was similar (for example, 50%/50%, 60%/40%), or generated a whole length of the more abundant subpopulation when the abundance of the two subpopulations was distinct enough (for example, 70%/30%, 80%/20%, 90%/10%). In some cases, metaSPAdes and MEGAHIT also generated the variation region as a contig of a less abundant subpopulation.

For three subpopulations, four different cases were tested with simulated genomes and reads. (1) In the first case, the starting and the ending sites of the variation region among the three subpopulations are the same (Supplementary Fig. 5a). No matter what the sequencing depths of the three subpopulations are, IDBA_UD always broke at the point of the variation starting and the ending sites, generating 5 contigs. While metaSPAdes obtained 5 contigs only when the sequencing depths were 50 X for all three subpopulations, in other assemblies, it obtained one full genome of the most dominant one, or three contigs (including the full genome of the most dominant one, each of the other two contigs represented the variable region of a less dominant subpopulation). For MEGAHIT, it obtained 5 contigs that broke at the point of the variation starting and the ending sites, in four of six of the tested sequencing depths combinations. In the other two sequencing depth combinations, MEGAHIT did the same as metaSPAdes, generating three contigs. (2) In the second case, the variation region starts at different sites and ends at the same site (which is the same end at different sites but starts at the same site, thus not shown here) (Supplementary Fig. 5b). (3) In the third and fourth cases (Supplementary Figs. 5c and d), the variation region starts at different sites and also ends at different sites.

From the assemblies in these three cases, we could obviously conclude that, no matter what the sequencing depth combination is, IDBA_UD always broke at the variation points, generating the same number of contigs for all the assemblies in each of the three cases.

COBRA accurately joins viral sequences from a simulated soil dataset. To evaluate the accuracy of COBRA in joining assembled sequences from more complicated ecosystems, we tested it on a composite soil metagenome dataset (See Supplementary methods for details). The dataset contained ~20 Gb reads *in silico* sequenced from 4,400 simulated soil viral genomes, and ~12 Gb reads from a natural soil metagenomic dataset. The composite dataset was assembled, and assembled scaffolds ≥ 2.5 kbp were filtered based on BLASTn search and used as queries for subsequent COBRA analyses (Extended Data Fig. 4a).

A total of 7,532 scaffolds were subjected to COBRA analyses, these scaffolds had an average length of 36.8 kbp (2.5-480 kbp), and an average depth of 49X (5-284X) (Extended Data Figs. 4b-d). COBRA identified 341 circular genomes (i.e., self_circular), and joined 2,413 of them as partial genomes (i.e., extended_partial), and 136 of them as circular genomes (i.e., extended_circular) (Extended Data Fig. 4e). As observed for the ocean virome dataset, we found the "orphan_end" queries usually had significantly lower coverage than those in the other COBRA categories (Extended Data Fig. 5a). The high alignment fraction of the extended COBRA sequences (Extended Data Fig. 5b) and the high similarity with the corresponding simulated genomes (Extended Data Fig. 5c), collectively documented the accuracy of COBRA when applied to this soil metagenomic dataset. Notably, COBRA raised the average length of scaffolds in the categories of "extended_circular" and "extended_partial" by > 30 kbp via joining up to 19 scaffolds (Extended Data Figs. 5d and e).

We also compared the performance of COBRA against that of binning tools (Extended Data Fig. 6) and found that COBRA obtained the highest fraction of good sequences (93.7% v.s. 15.4-79.7%), with the longest average length (62 kbp v.s. 48-53 kbp), and the lowest rate of contaminated sequences (0.4% v.s., 13.6-83.3%). Although metaBAT2 obtained the longest total sequence of good bins (60 Mb v.s. 8-42 Mb), the relatively high rate of contaminated bins implies inaccuracy.

Performance comparison of COBRA against ContigExtender. ContigExtender is a tool using a novel recursive extending strategy that could explore multiple extending paths to achieve highly accurate longer contigs using input reads [30]. The primary difference between COBRA and ContigExtender is that, the former extends the target contigs by joining with other assembled contigs, while the latter does that via using the consensus sequences of retrieved reads mapped to the end of the target contigs. Here we compare their performance using the same contig set we used for the comparison between COBRA and the binning tools. We could only compare the results for 29 contigs, given the extremely slow processing of ContigExtender (Supplementary Table 4). COBRA identified 6 self_circular contigs, extended 6 contigs to circular genomes, and another 4 contigs to partial genomes, with an extended length of 374-17,098 bp and 100% accuracy. 11 were orphan_end contigs (average coverage, 13X) and 2 were extended_failed contigs (average coverage,

18X). ContigExtender extended all but one of the contigs (it provides no information about circularization), with less extended length (1-7,779 bp) than COBRA and with much lower average accuracy (55%).

Our comparison of COBRA and ContigExtender suggested that the latter tool could extend contigs with low sequencing coverage but the program is very slow, and sometimes makes incorrect extensions (Supplementary Table 4). Thus, we suggest that ContigExtender could be utilized in some specific circumstances but it is not ideal for a large dataset.

Expansion of the diversity of actinophages with *whiB* family transcriptional regulators. We assessed the impact of our phage genome collection on the sampled diversity of another group other than the huge phages. Actinobacteria are abundant in the studied ecosystems, and one of their infecting phage groups could be determined via the identification of the *whiB* regulators [44]. In fact, 477 of the 7,334 new phage genomes encoded *whiB* (thus assigned as actinophages). The phylogeny based on concatenated sequences of core structure proteins of actinophages from the current study and published sources revealed that several subclades were defined using genomes from this study (Supplementary Fig. 9a). Notably, members of certain clades appeared to encode two or more copies of *whiB* genes. Regardless of the source of genome data, actinophage genomes, both with and without the *WhiB* gene, exhibit a wide range of sizes, although only a few of them are huge phages (Supplementary Fig. 9b).

The newly available high-quality actinophage genomes from Lake Rotsee enabled RNA analyses of a large enough set of phages to be able to detect differences in expression patterns of different gene types. Most of the *whiB* genes were either inactive or transcribed at very low levels at the time of sampling. The majority of the active phages were in the late replication stage, with core structural protein-coding genes (e.g., major capsid) being expressed (Supplementary Fig. 10). In one active phage encoding two *whiB* genes, we observed distinct transcription levels for each gene, which likely indicates different roles for these genes in the phage's life cycle.

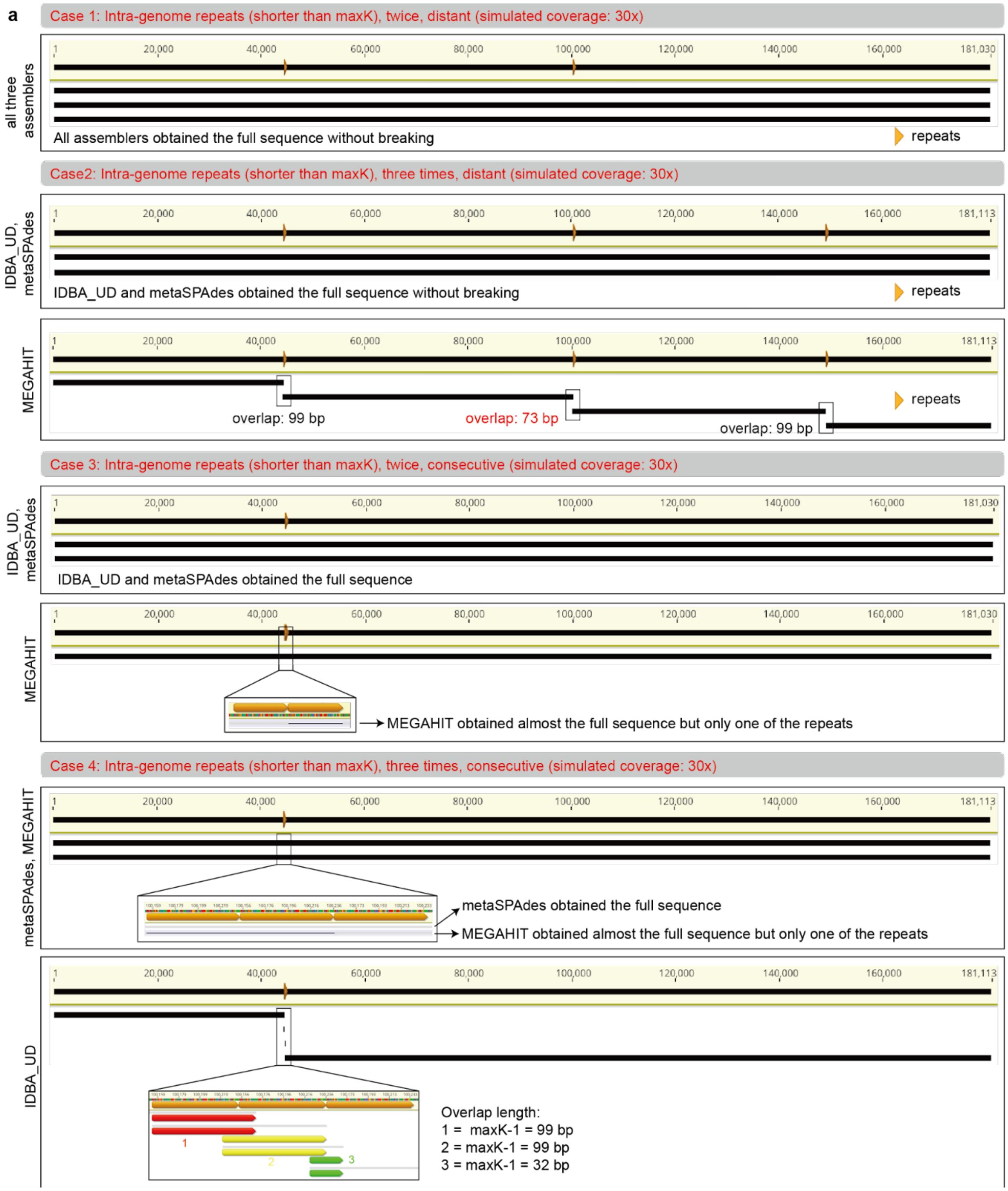

Supplementary Fig. 1 | Simulated sequences with intra-genome repeats shorter than the largest kmer used in *de novo* assembly. Different cases include: (case 1) repeats occurring twice and distant from each other, (case 2) repeats occurring triple times and distant from each other, (case 3) repeats occurring twice and consecutive with each other, and (case 4) repeats occurring triple times and consecutive with each other. The simulated genomes were in silico "sequenced" with a coverage of 30X, and the generated reads were assembled using metaSPAdes, IDBA_UD, and MEGAHIT in each case. If a given assembly obtained more than one contig, the contigs' overlapped sequence lengths (if detected) are shown.

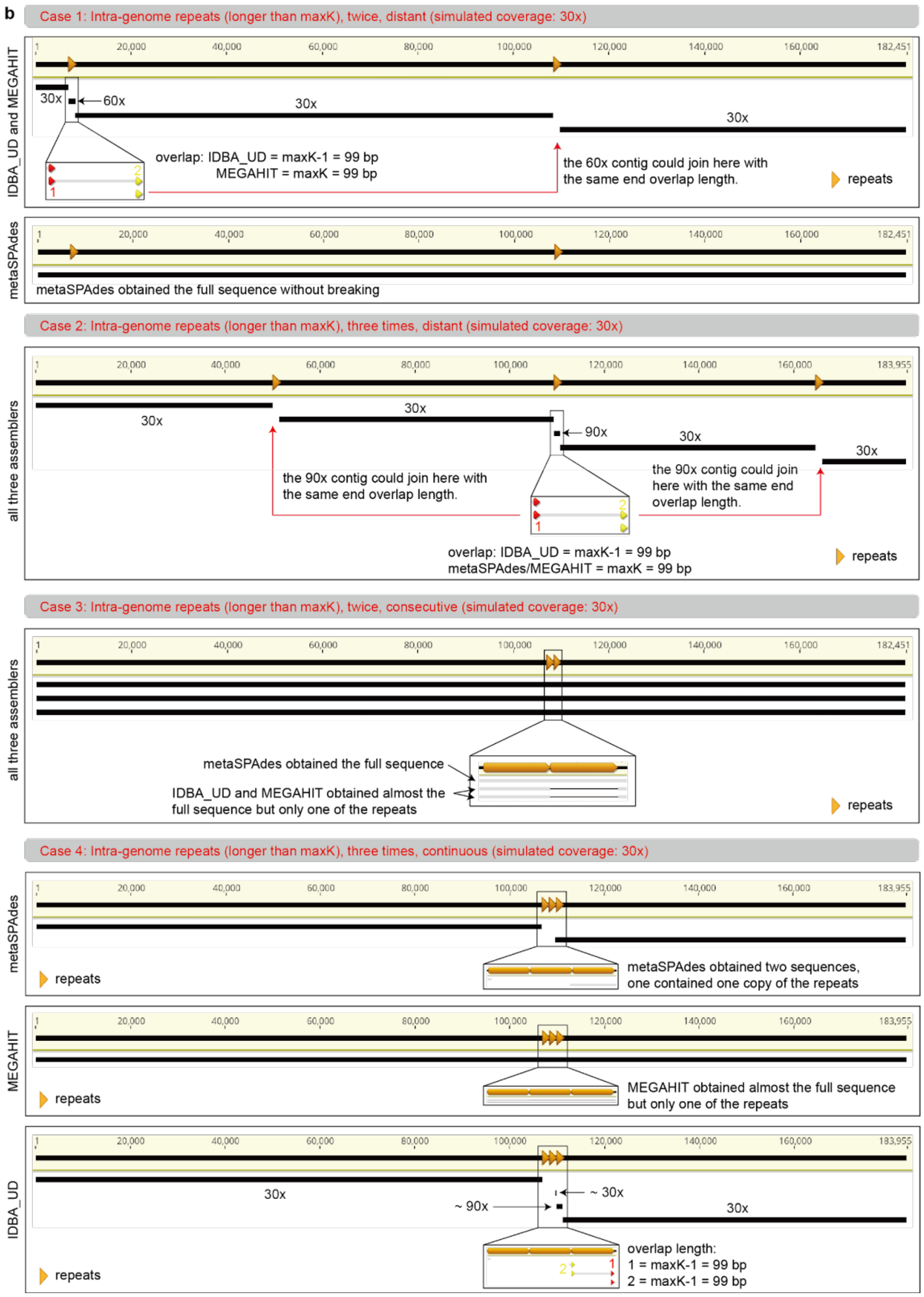

Supplementary Fig. 2 | Simulated sequences with intra-genome repeats that are longer than the largest kmer used in *de novo* assembly. Different cases include: (case 1) repeats occurring twice and distant from each other, (case 2) repeats occurring triple times and distant from each other, (case 3) repeats occurring twice and consecutive with each other, and (case 4) repeats occurring triple times and consecutive with each other. The simulated genomes were in silico "sequenced" with a coverage of 30X, and the generated reads were assembled using metaSPAdes, IDBA_UD, and MEGAHIT in each case. If a given assembly obtained more than one contig, the overlapped sequence lengths (if detected) of the contigs are shown.

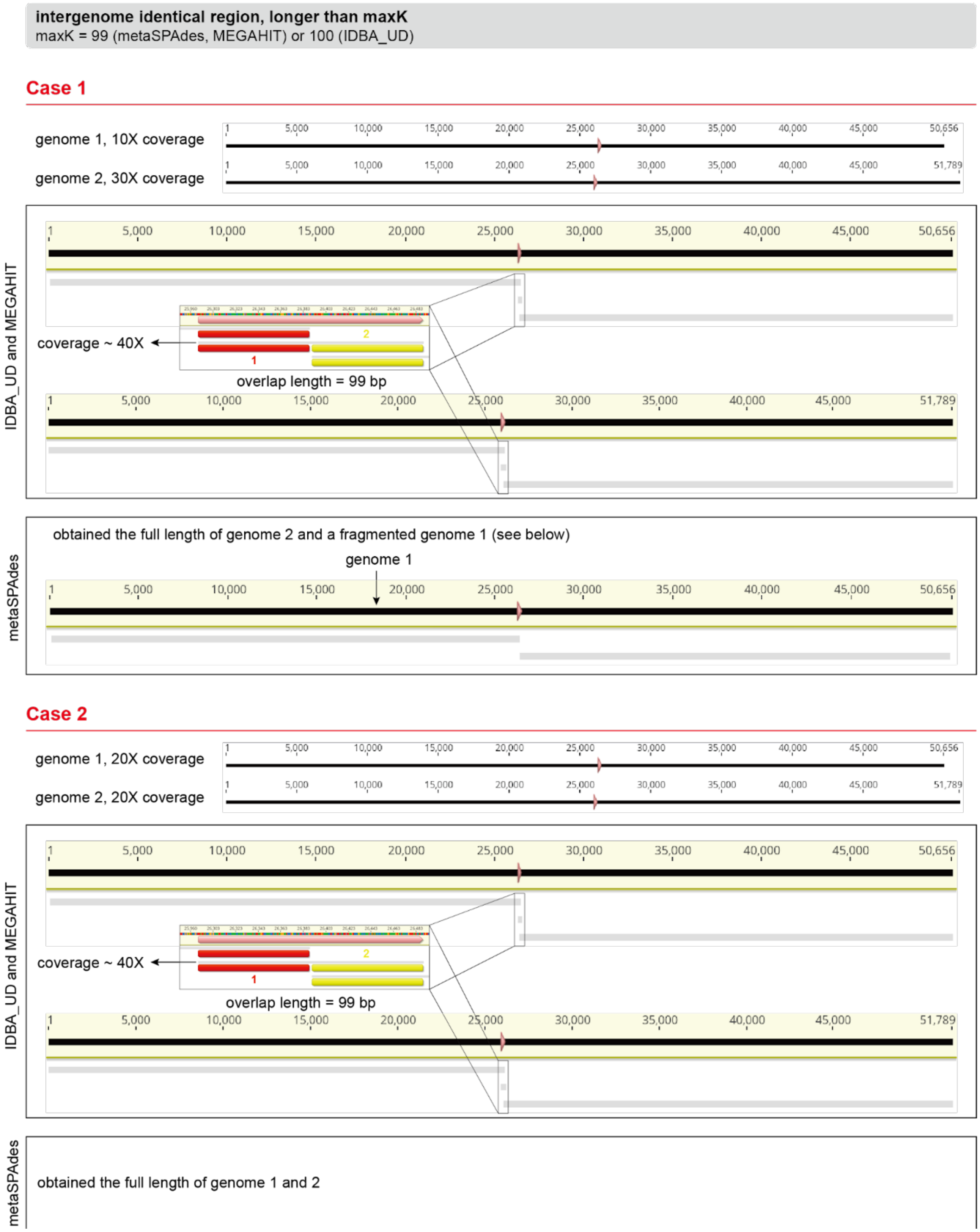

Supplementary Fig. 3 | Simulated sequences with inter-genome shared regions. Two different cases are shown: (case 1) where the two simulated genomes were in silico "sequenced" with coverage of 10X (genome 1) and 30X (genome 2), and (case 2) where both of the two simulated genomes were in silico "sequenced" with coverage of 20X. The generated reads were assembled using metaSPAdes, IDBA_UD, and MEGAHIT in each coverage combination. If a given assembly obtained more than one contig, the contigs' overlapped sequence lengths (if detected) are shown.

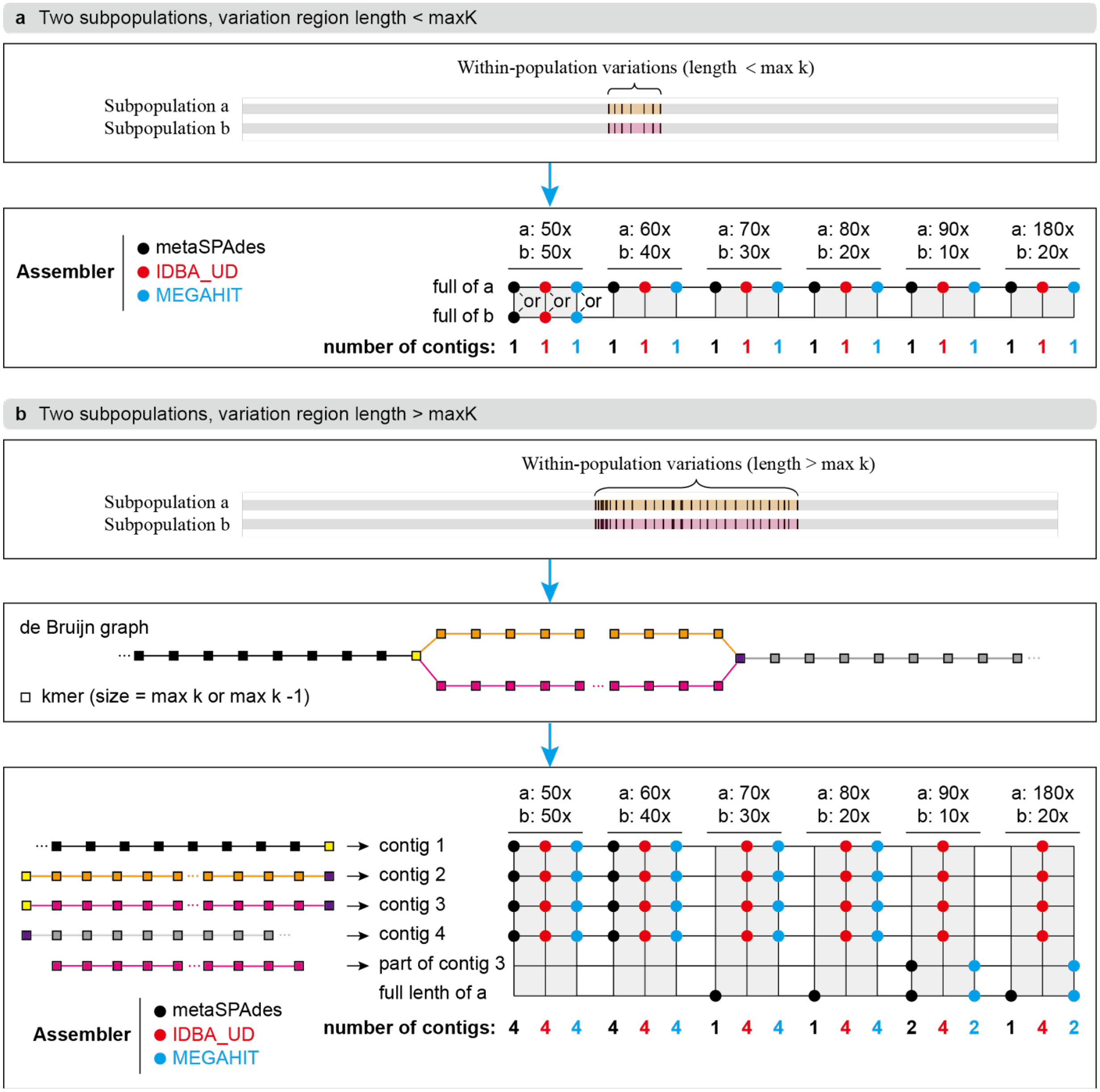

Supplementary Fig. 4 | Simulated sequences with variations between two subpopulations. The length of the variation region between the two subpopulations is compared for two cases: (a) when it is shorter than the maximum kmer (maxK) used in the assembly, and (b) when it is longer than the maxK used in the assembly. The simulated genomes were in silico "sequenced" with different coverage combinations, and the generated reads were assembled using metaSPAdes, IDBA_UD, and MEGAHIT in each coverage combination. The resulting contigs for each coverage combination are represented by colored circles.

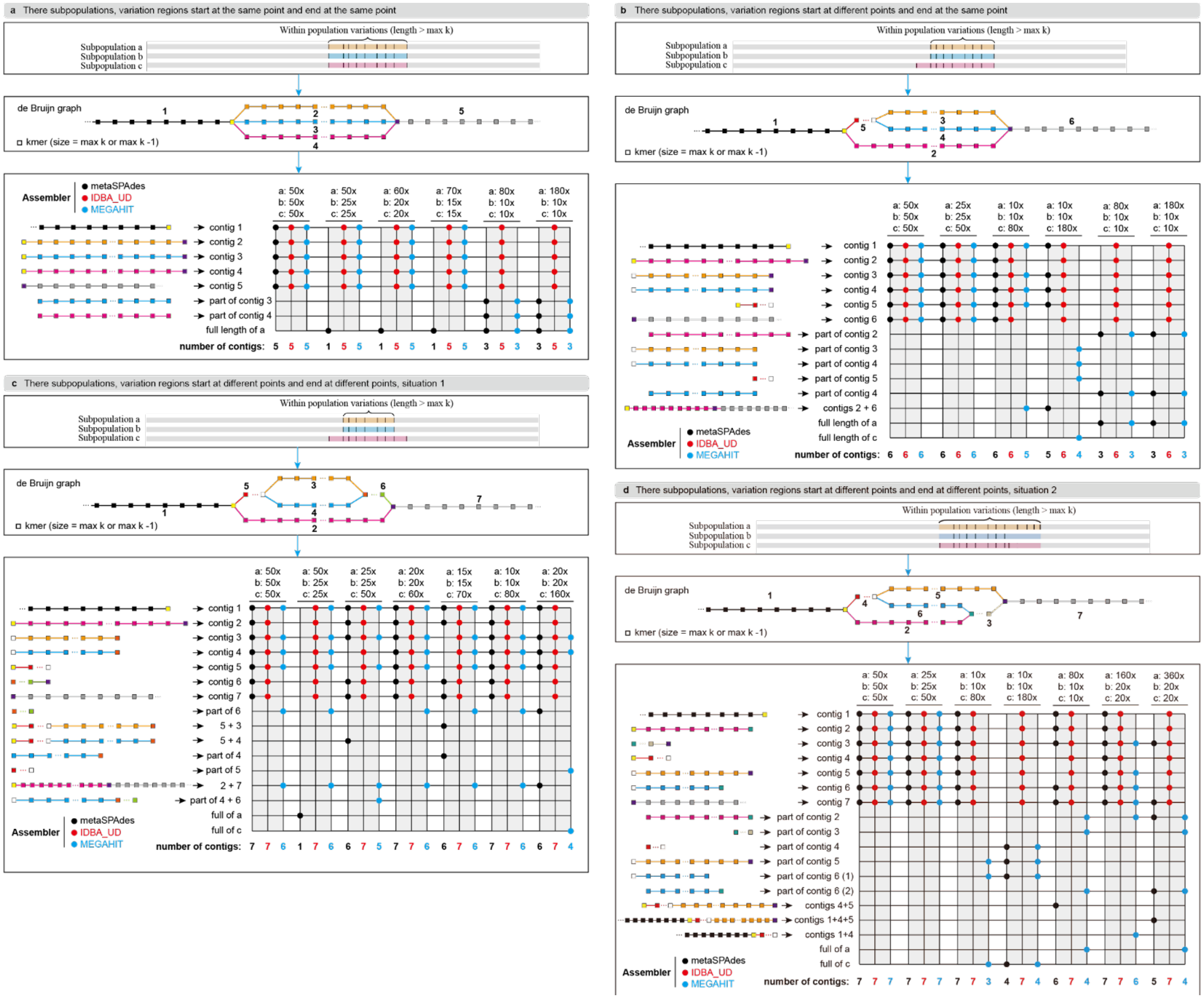

Supplementary Fig. 5 | Simulated sequences with variations among three subpopulations. The simulated genomes contain variation regions within three subpopulations, characterized by (a) identical start and end points, (b) different start points but the same endpoint, (c) different start and end points (situation 1), and (d) different start and end points (situation 2). The length of the variation regions exceeds the maximum kmer (maxK) used in the de novo assembly. The genomes were in silico "sequenced" with different coverage combinations, and the resulting reads were individually assembled using metaSPAdes, IDBA_UD, and MEGAHIT for each coverage combination. The contigs obtained from each coverage combination are represented by colored circles.

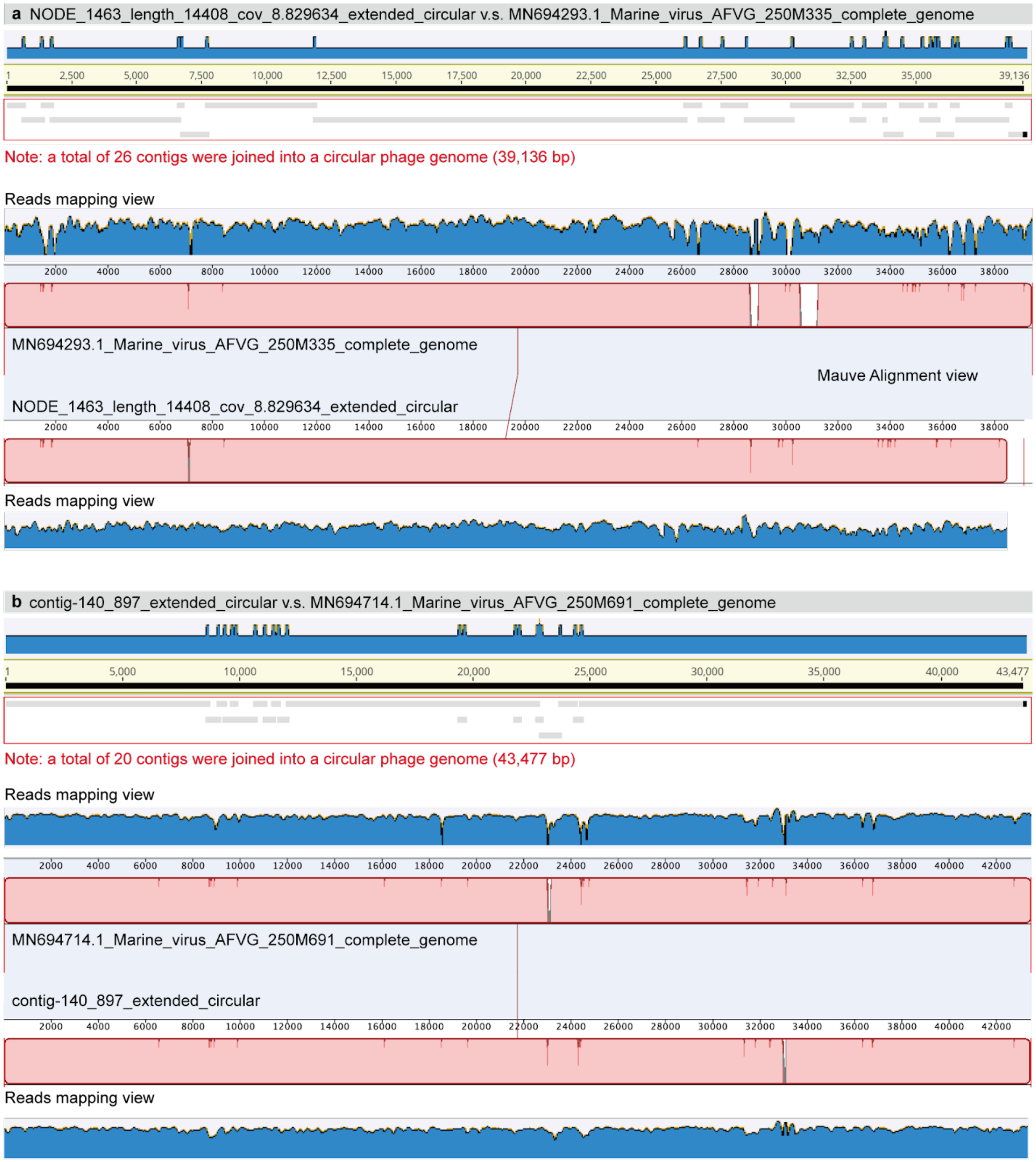

Supplementary Fig. 6 | Circular extension of query virus contigs by COBRA. Illustrative examples of genomes joined by COBRA that exhibit similarity to a polished genome, with contigs assembled using (a) metaSPAdes and (b) IDBA_UD. The Mauve Alignment view showcases the comparison between the COBRA "extended_circular" genome and the corresponding polished genome, along with their respective read mapping profiles for validation.

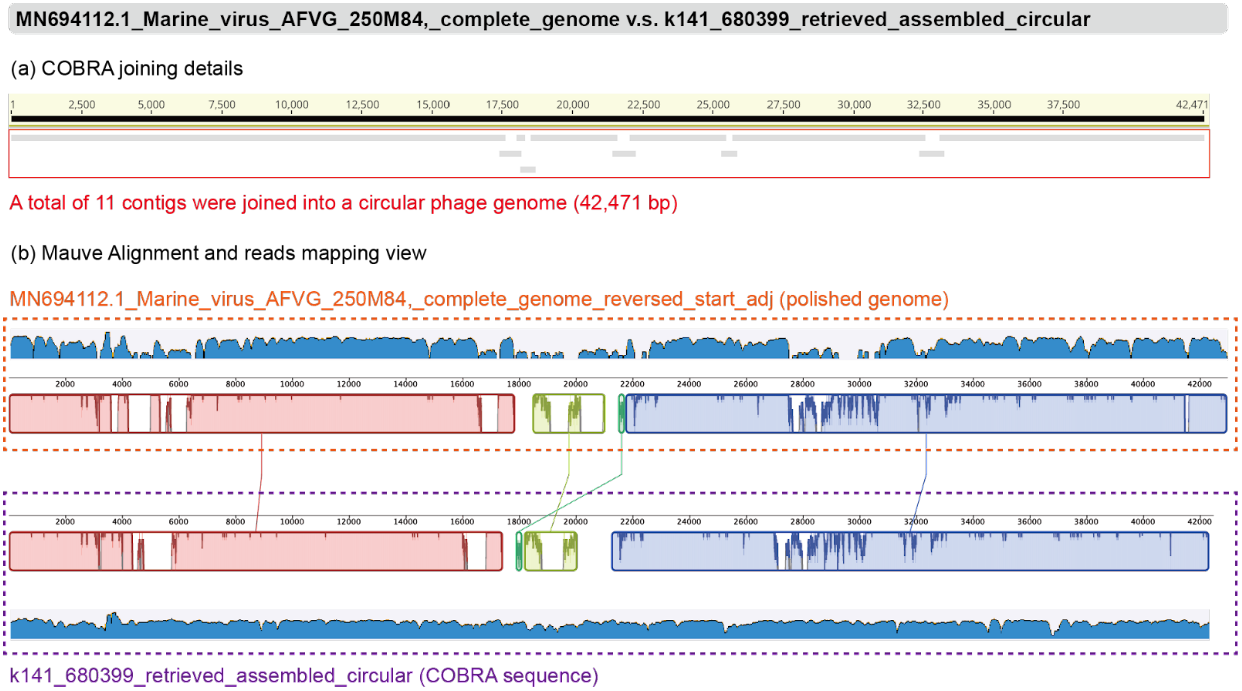

Supplementary Fig. 7 | One "extended_circular" from the MEGAHIT assembly with < 90% "AF_COBRA" and < 90% "AF_polished". (a) The original contig was assembled by MEGAHIT, which was extended to a circular genome including a total of 11 contigs. (b) The mauve alignment and reads mapping profiles show that the COBRA genome represents the dominant subpopulation while the polished genome represents the rare subpopulation in the sample.

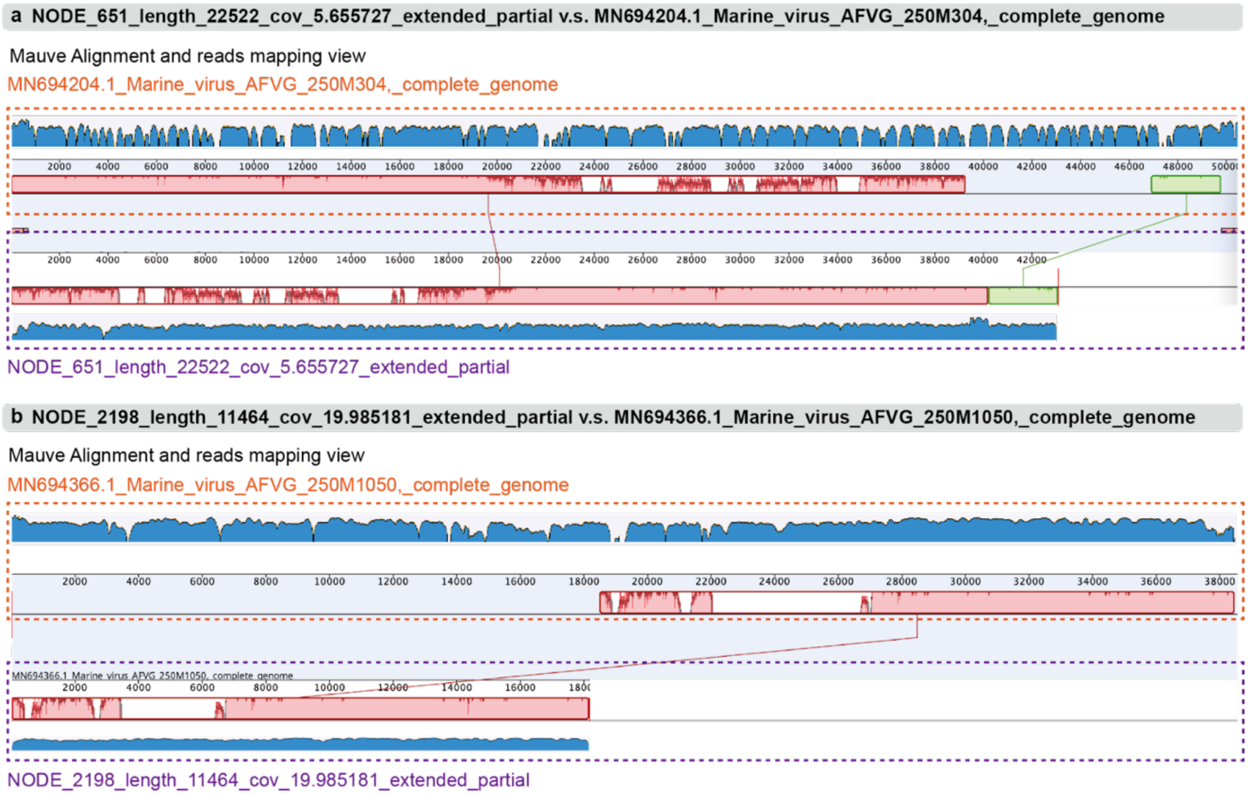

Supplementary Fig. 8 | Examples of COBRA and polished genome pairs with relatively lower "AF_COBRA" values. To document that such pairs are due to variations between subpopulations, the mauve alignment and read mapping profiles for each pair of the polished genome and COBRA sequence are shown.

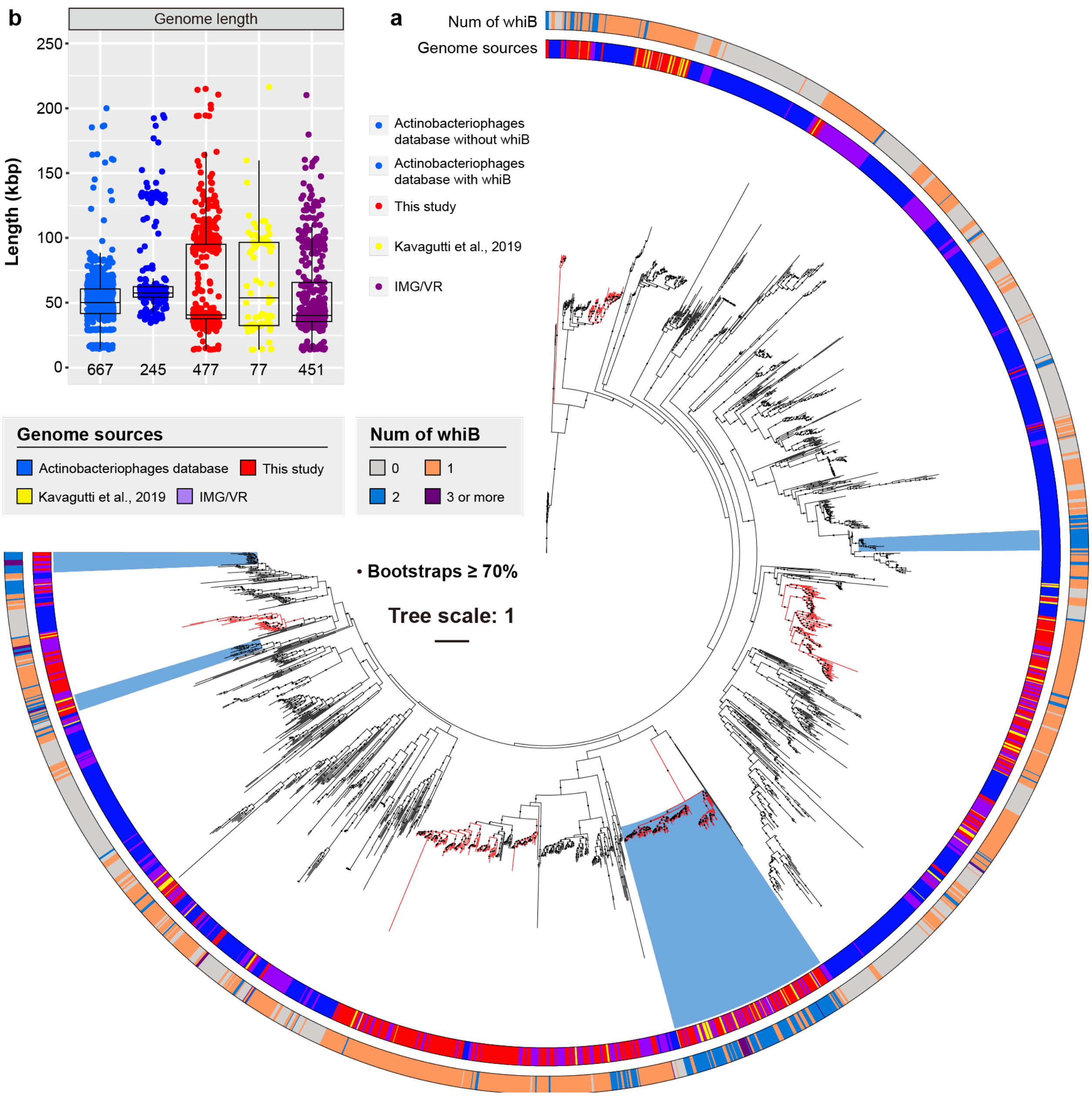

Supplementary Fig. 9 | Freshwater ecosystem genomes broaden the diversity of *whiB*-encoding actinophages. (a) Phylogeny of actinophage genomes based on the concatenated sequences of core structural proteins including *TerL*. The numbers of *whiB* genes in genomes are displayed by colored stripes in the outer ring. The sources of the genomes are denoted by colored stripes in the inner ring. Subclades predominantly featuring genomes from this study are highlighted in red. Subclades, where the majority of genomes encode two or more *whiB* genes, are emphasized in light blue. (b) The length distribution of the genomes with or without the *whiB* gene. The number of genomes from each category is shown below each bar. In the box plot, the centre lines, upper and lower bounds, and upper and lower whiskers show median values, 25th and 75th quantiles, and the largest and smallest non-outlier values, respectively. Outliers are defined as having a value >1.5 × interquartile range (IQR) away from the upper or lower bounds.

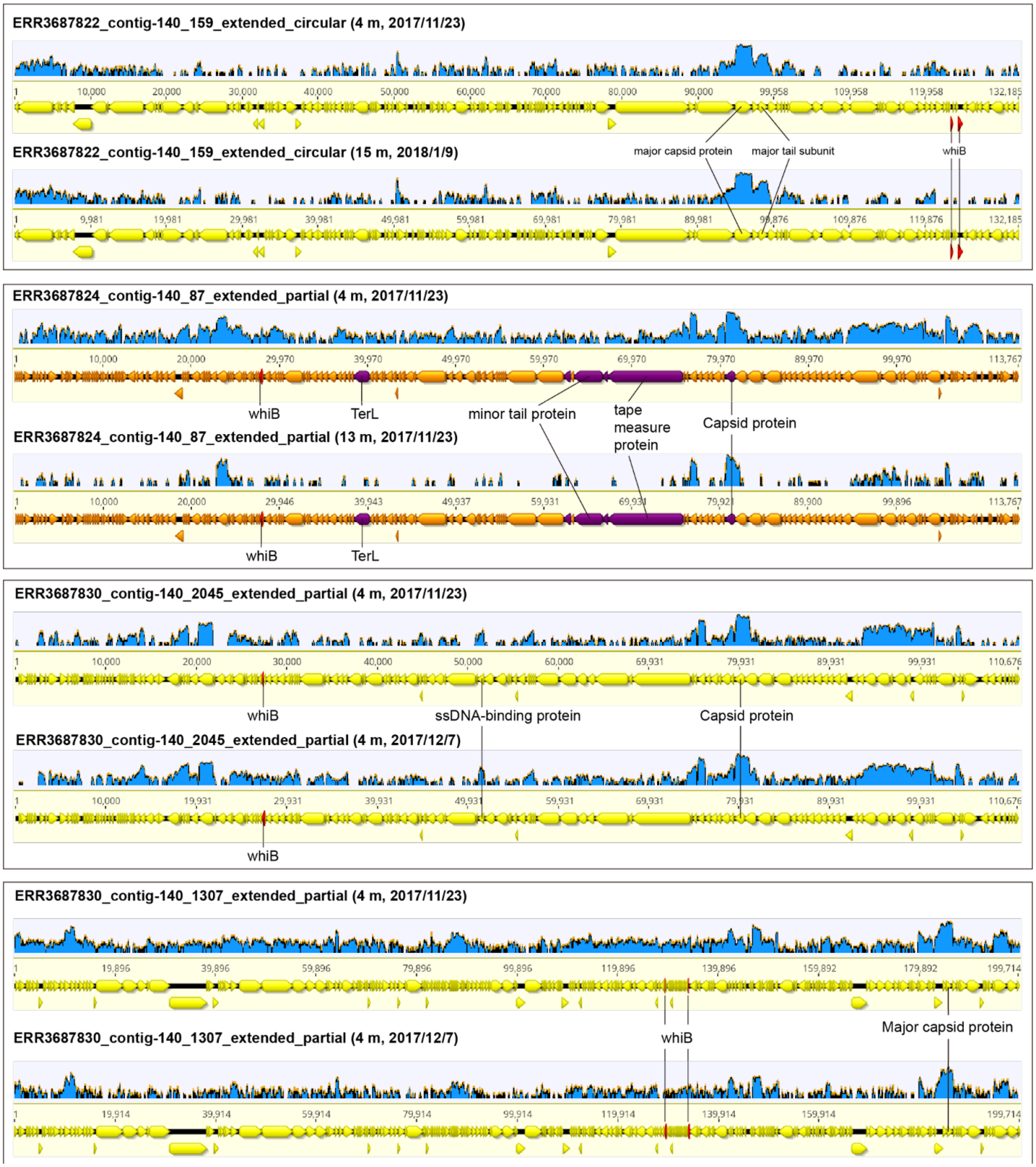

Supplementary Fig. 10 | Examples of the *in situ* gene expression of whiB-encoding actinophages with genomes reconstructed from Lake Rotsee. The reads from different metatranscriptomic samples were mapped to each of the four genomes using Bowtie2 with default parameters. Then the bam files were imported into Geneious and remapped allowing no mismatch for each read. The whiB genes are highlighted in red, some core phage structural protein genes are indicated with their annotations shown. The genome names, the sampling depths, and the sampling dates of the corresponding transcriptomic sample are shown at the top of each profile. A total of four actinophage genomes are shown (the one with two whiB genes is shown at the bottom), with each profiled with two transcriptomic samples.

(a) Zhao et al., 2022, HugePhage 1, 852 kbp

- evaluated by viral identification tools

CheckV: viral_genes = 33, host_genes = 533, provirus = yes, termini = complete-prophage, viral_length = 67,045 bp, viral region = 487042-554086

VIBRANT: viral length = 84,279 bp, life style = lysogenic, viral region = ~490936 - ~584027 bp

- alignment with contigs from Delftia acidovorans strain 2167 DR66.Contig240 (a bacterial genome; NCBI ID = JOUB01000003)

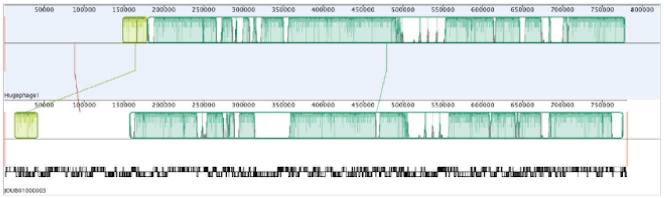

Based on all the information we obtained here, the HugePhage 1 sequence contains a prophage genome (67-84 kbp in length), given the genome wide alignment with the contigs of a Delftia acidovorans genome from NCBI, and the high similarity of large terminase protein with those from Delftia spp., we conclude that "HugePhage 1" should be a small phage of Delftia member.

- high similarity of HugePhage 1 large terminase to those of Delftia spp.

Description	Scientific Name	Max Score	Total Score	Query Cover	E value	Per. Ident	Acc. Len	Accession
terminase family protein [Delftia sp. ZNC0008]	Delftia sp. ZNC0008	1040	1040	100%	0.0	99.42%	515	WP_047469065.1
terminase family protein [Delftia sp. RIT313]	Delftia sp. RIT313	1040	1040	100%	0.0	99.42%	515	WP_043821036.1
terminase family protein [Delftia sp. ZNC0008]	Delftia sp. ZNC0008	1035	1035	100%	0.0	98.64%	515	WP_047473737.1
terminase family protein [Delftia tsuruhatensis]	Delftia tsuruhatensis	1034	1034	100%	0.0	98.45%	515	WP_046984607.1
terminase family protein [Delftia acidovorans]	Delftia acidovorans	1033	1033	100%	0.0	98.45%	515	WP_034395113.1
terminase family protein [Delftia acidovorans]	Delftia acidovorans	1033	1033	100%	0.0	98.45%	515	WP_097203122.1
terminase [Delftia acidovorans]	Delftia acidovorans	1032	1032	100%	0.0	98.45%	515	WP_239971178.1
terminase family protein [Delftia tsuruhatensis]	Delftia tsuruhatensis	1032	1032	100%	0.0	98.45%	515	WP_133073502.1
terminase [Delftia acidovorans]	Delftia acidovorans	1032	1032	100%	0.0	98.25%	515	PJO37630.1
terminase family protein [Delftia tsuruhatensis]	Delftia tsuruhatensis	1032	1032	100%	0.0	98.25%	515	WP_046239682.1
terminase family protein [Delftia acidovorans]	Delftia acidovorans	1030	1030	100%	0.0	98.25%	515	WP_180319372.1
terminase family protein [Delftia acidovorans]	Delftia acidovorans	1029	1029	100%	0.0	98.06%	515	MBN9323155.1
terminase family protein [Delftia acidovorans]	Delftia acidovorans	1028	1028	100%	0.0	98.06%	515	WP_197956440.1
terminase family protein [Delftia sp. SD018]	Delftia sp. SD018	1028	1028	100%	0.0	98.06%	515	WP_207475865.1

(b) Zhao et al., 2022, HugePhage 2, 839 kbp

- evaluated by viral identification tools

CheckV: viral_genes = 37, host_genes = 533, provirus = yes, termini = complete-prophage, viral_length = 27,719 bp, viral region = 154998-182716

VIBRANT: viral length = 32,493 bp, life style = lysogenic, viral region = ~148362 - ~183490 bp

- alignment with contigs from Ruminococcus sp. CAG:108 (a bacterial genome; NCBI ID = CAXD010000000)

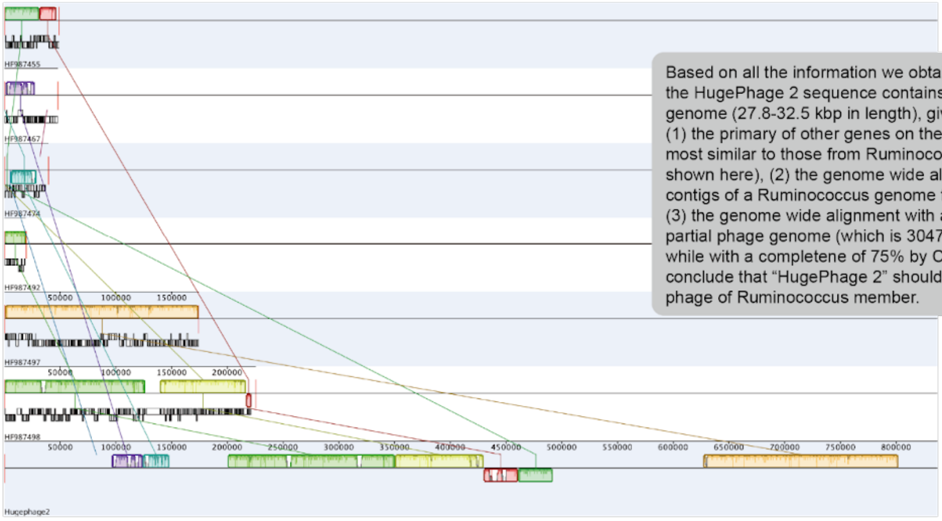

Based on all the information we obtained here, the HugePhage 2 sequence contains a prophage genome (27.8-32.5 kbp in length), given that (1) the primary of other genes on the sequence were most similar to those from Ruminococcus sp. (not shown here), (2) the genome wide alignment with the contigs of a Ruminococcus genome from NCBI, and (3) the genome wide alignment with a published partial phage genome (which is 30475 bp in length, while with a completene of 75% by CheckV), we conclude that "HugePhage 2" should be a small phage of Ruminococcus member.

- alignment with the partial genome of Caudoviricetes sp. isolate ctxHS4 (NCBI ID = BK019798), whose host is Ruminococcus (Tisza and Buck, 2021)

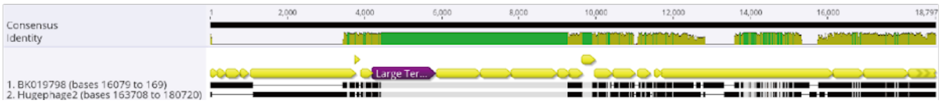

see the text legend on the next page.

Supplementary Fig. 11 | Evidence indicating that the two published huge phages with genome sizes over 800 kbp were prophage sequences. (a) HugePhage 1 and (b) HugePhage 2 reported by Zhao et al. (Zhao et al. 2022). The genomes were downloaded via the link provided in the corresponding literature, and evaluated using the viral identification tools of CheckV (Nayfach et al. 2020) and VIBRANT (Kieft et al. 2020) to obtain the related information, including "viral_length", "viral region", "life style" and others. The genomes were searched against NCBI for highly similar sequences, which led to the identification of the corresponding bacterial genomes (and one phage genome for HugePhage 2) as shown in the figure. The protein-coding genes predicted using Prodigal (-m -p single) (Hyatt et al. 2010) from the two phage genomes were searched against the RefSeq database (Pruitt et al. 2007) using BLASTp for their taxonomic assignment. According to the corresponding literature of Caudoviricetes sp. isolate ctxHS4 (NCBI ID = BK019798), i.e., Tisza and Buck (Tisza and Buck 2021), the bacterial host of this phage is predicted as "Ruminococcus".

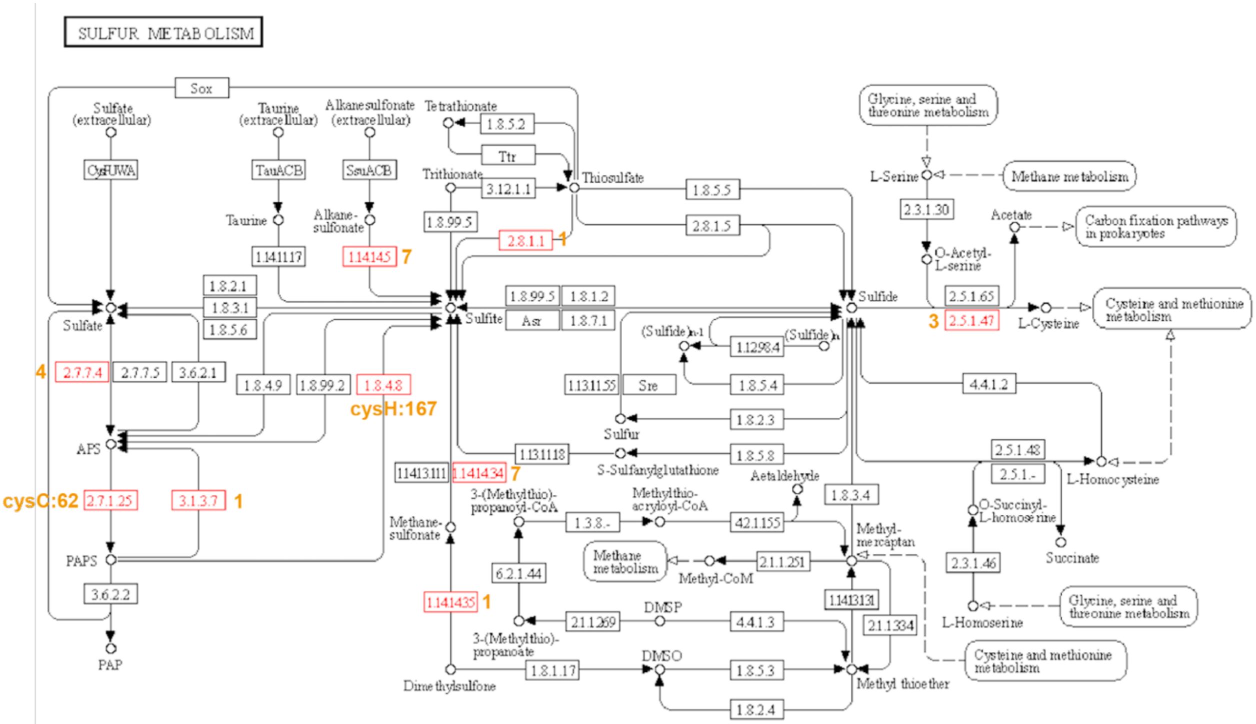

Supplementary Fig. 12 | The number of sulfur metabolism-related AMGs detected in the phage genomes. The detected genes are highlighted in red, and the corresponding number of genes is shown.
