# Supplementary material for: COBRA improves the completeness and contiguity of viral genomes assembled from metagenomes
Source: Nat Microbiol. 2024 Feb 6;9(3):737–50. doi: 10.1038/s41564-023-01598-2 (PMC10914622; doi:10.1038/s41564-023-01598-2)
Supplement: Supplementary file 2 — Reporting Summary [file 41564_2023_1598_MOESM2_ESM.pdf]

nature portfolio

Corresponding author(s): Linxing Chen & Jillian F Banfield

Last updated by author(s): Dec 5, 2023

Reporting Summary

Nature Portfolio wishes to improve the reproducibility of the work that we publish. This form provides structure for consistency and transparency in reporting. For further information on Nature Portfolio policies, see our Editorial Policies and the Editorial Policy Checklist.

Statistics

For all statistical analyses, confirm that the following items are present in the figure legend, table legend, main text, or Methods section.

n/a	Confirmed	
☐	☒	The exact sample size (*n*) for each experimental group/condition, given as a discrete number and unit of measurement
☒	☐	A statement on whether measurements were taken from distinct samples or whether the same sample was measured repeatedly
☐	☒	The statistical test(s) used AND whether they are one- or two-sided<br>*Only common tests should be described solely by name; describe more complex techniques in the Methods section.*
☒	☐	A description of all covariates tested
☒	☐	A description of any assumptions or corrections, such as tests of normality and adjustment for multiple comparisons
☐	☒	A full description of the statistical parameters including central tendency (e.g. means) or other basic estimates (e.g. regression coefficient) AND variation (e.g. standard deviation) or associated estimates of uncertainty (e.g. confidence intervals)
☐	☒	For null hypothesis testing, the test statistic (e.g. *F*, *t*, *r*) with confidence intervals, effect sizes, degrees of freedom and *P* value noted<br>*Give P values as exact values whenever suitable.*
☒	☐	For Bayesian analysis, information on the choice of priors and Markov chain Monte Carlo settings
☒	☐	For hierarchical and complex designs, identification of the appropriate level for tests and full reporting of outcomes
☒	☐	Estimates of effect sizes (e.g. Cohen's *d*, Pearson's *r*), indicating how they were calculated

Our web collection on statistics for biologists contains articles on many of the points above.

Software and code

Policy information about availability of computer code

Data collection	sratoolkit.2.11.1 is used to download the SRA reads from NCBI.
Data analysis	metaSPAdes v3.15.5, IDBA_UD, MEGAHIT v1.2.9, Geneious Prime, InSilicoSeq, sickle version 1.33, bbmap Version 39.01, blastn: 2.14.0+, Bowtie2 version 2.3.5.1, MetaBAT version 2.12.1, fastANI version 1.3, checkV version 0.7.0, vRhyme Version 1.1.0, CoCoNet, VIBRANT v1.2.1, Prodigal V2.6.3, hmmsearch version HMMER 3.3, IQ-TREE version 1.6.12, MAFFT v7.453, trimAL v1.4.rev15, ContigExtender, genomad, MUSCLE version 5.1.linux64, COBRA version1.2.2

For manuscripts utilizing custom algorithms or software that are central to the research but not yet described in published literature, software must be made available to editors and reviewers. We strongly encourage code deposition in a community repository (e.g. GitHub). See the Nature Portfolio guidelines for submitting code & software for further information.

Data

Policy information about availability of data

All manuscripts must include a data availability statement. This statement should provide the following information, where applicable:

- Accession codes, unique identifiers, or web links for publicly available datasets
- A description of any restrictions on data availability
- For clinical datasets or third party data, please ensure that the statement adheres to our policy

The high-quality and complete genomes obtained from the 231 freshwater metagenomes are available at figshare via https://figshare.com/articles/dataset/viral_genomes_fasta/23282789. The Actinobacteriophage database used in this study is available at https://phagesdb.org/.

Research involving human participants, their data, or biological material

Policy information about studies with human participants or human data. See also policy information about sex, gender (identity/presentation), and sexual orientation and race, ethnicity and racism.

Reporting on sex and gender	Not applicable.
Reporting on race, ethnicity, or other socially relevant groupings	Not applicable.
Population characteristics	Not applicable.
Recruitment	Not applicable.
Ethics oversight	Not applicable.

Note that full information on the approval of the study protocol must also be provided in the manuscript.

Field-specific reporting

Please select the one below that is the best fit for your research. If you are not sure, read the appropriate sections before making your selection.

☐ Life sciences ☐ Behavioural & social sciences ☒ Ecological, evolutionary & environmental sciences

For a reference copy of the document with all sections, see nature.com/documents/nr-reporting-summary-flat.pdf

Ecological, evolutionary & environmental sciences study design

All studies must disclose on these points even when the disclosure is negative.

Study description	This study develops a tool that identify the fragmentation points of de bruijn graph based short reads assemblers and join the contigs if some specific conditions are met.
Research sample	A total of 231 published freshwater metagenomes were included in analyses, for each of them, their paired reads were downloaded from NCBI, followed by quality control, de novo assembly, viral contig prediction and analyses by the tool developed in this study.
Sampling strategy	We analyzed 231 published freshwater metagenomes from different countries around the world, which is sufficient to test the performance of the tool developed in this study.
Data collection	na
Timing and spatial scale	na
Data exclusions	na
Reproducibility	na
Randomization	na
Blinding	na

Did the study involve field work? ☐ Yes ☒ No

Reporting for specific materials, systems and methods

We require information from authors about some types of materials, experimental systems and methods used in many studies. Here, indicate whether each material, system or method listed is relevant to your study. If you are not sure if a list item applies to your research, read the appropriate section before selecting a response.

Materials & experimental systems

n/a	Involved in the study
☒	☐ Antibodies
☒	☐ Eukaryotic cell lines
☒	☐ Palaeontology and archaeology
☒	☐ Animals and other organisms
☒	☐ Clinical data
☒	☐ Dual use research of concern
☒	☐ Plants

Methods

n/a	Involved in the study
☒	☐ ChIP-seq
☒	☐ Flow cytometry
☒	☐ MRI-based neuroimaging
